# Country data on AMR in Pakistan in the context of community-acquired respiratory tract infections: links between antibiotic susceptibility, local and international antibiotic prescribing guidelines, access to medicine and clinical outcome

**DOI:** 10.1093/jac/dkac213

**Published:** 2022-09-06

**Authors:** Didem Torumkuney, Bushra Jamil, Summiya Nizamuddin, James van Hasselt, Uzma Pirzada, Rendani Manenzhe

**Affiliations:** GlaxoSmithKline, 980 Great West Road, Brentford, Middlesex TW8 9GS, UK; Department of Medicine, Section of Infectious Diseases, The Aga Khan University Hospital, Karachi, Pakistan; Shaukat Khanum Memorial Cancer Hospital and Research Centre, Section of Microbiology, Department of Pathology, 7A Block R3, Johar Town, Lahore, Pakistan; GlaxoSmithKline, The Campus, Flushing Meadows, 57 Sloane Street, Bryanston, Gauteng, 2021, South Africa; GlaxoSmithKline, 35 Dockyard Road, West Wharf, Karachi 74000, Pakistan; GlaxoSmithKline, The Campus, Flushing Meadows, 57 Sloane Street, Bryanston, Gauteng, 2021, South Africa

## Abstract

**Background:**

Antimicrobial resistance (AMR) is one of the biggest threats to global public health. Selection of resistant bacteria is driven by inappropriate use of antibiotics, amongst other factors. COVID-19 may have exacerbated AMR due to unnecessary antibiotic prescribing. Country-level knowledge is needed to understand options for action.

**Objectives:**

To review the current situation with respect to AMR in Pakistan and initiatives addressing it. Identifying areas where more information is required will provide a call to action to minimize any further rises in AMR and improve patient outcomes.

**Methods:**

National AMR initiatives, antibiotic use and prescribing in Pakistan, and availability of susceptibility data, in particular for the key community-acquired respiratory tract infection (CA-RTI) pathogens (*Streptococcus pneumoniae* and *Haemophilus influenzae*) were identified. National and international antibiotic prescribing guidelines for specific CA-RTIs (community-acquired pneumonia, acute otitis media and acute bacterial rhinosinusitis) commonly used locally were also reviewed, plus local antibiotic availability. Insights from a local clinician and clinical microbiologist were sought to contextualize this information.

**Conclusions:**

Pakistan is active in developing initiatives to address AMR such as compiling a National Action Plan. However, antibiotic consumption is high and although there is legislation in place prohibiting over-the-counter purchase of antibiotics, this is still possible. Healthcare professionals use local and international antibiotic prescribing guidelines for CA-RTIs when managing patients. As highlighted by the clinical microbiologist’s expert comments, surveillance of AMR in locally prevalent microorganisms is lacking. A more standardized inclusive approach in developing local guidelines, using up-to-date local surveillance data of isolates from community-acquired infections, could make management guideline use more locally relevant for clinicians. This would pave the way for a higher level of appropriate antibiotic prescribing and improved adherence. This would, in turn, potentially limit AMR development and improve clinical outcomes for patients.

## Introduction

Antimicrobial resistance (AMR) is one of the biggest threats to public health throughout the world,^1^ as described in the introductory paper of this Supplement.^2^ The WHO states that ‘the world urgently needs to change the way it prescribes and uses antibiotics. Even if new medicines are developed, without behaviour change, antibiotic resistance will remain a major threat’.^3^ The first paper in this Supplement included details about the multiple factors which can drive a rise in AMR, along with the global initiatives that are in place to address this threat.^2^ Each country and/or region is expected to play their part through local initiatives.

In order to identify how AMR can be addressed in Pakistan in the future, it is necessary to review what is happening now. In this paper, we present the current situation in Pakistan, determined by using published information (by searching PubMed, Google Scholar and other internet platforms) to ascertain any national initiatives to address AMR, antibiotic use and prescribing, and availability of susceptibility data, in particular for the key community-acquired respiratory tract infection (CA-RTI) pathogens *Streptococcus pneumoniae* and *Haemophilus influenzae*. National and international antibiotic prescribing guidelines for CA-RTIs [specifically community-acquired pneumonia (CAP), acute otitis media (AOM) and acute bacterial rhinosinusitis (ABRS)] commonly used by healthcare professionals in Pakistan were also reviewed, along with how these link to local antibiotic availability. Insights from a clinician and a clinical microbiologist were sought to contextualize this information. In addition, we aimed to identify areas where more information is required and present a call to action to improve clinical outcomes for patients and to minimize further rise in AMR within Pakistan.

## Action Plans

Following the formulation by the World Health Assembly in 2015 of a Global Action Plan (GAP) for AMR,^4^ many countries began to develop their own National Action Plan (NAP). The government of Pakistan formed an intra-sectoral committee on AMR in recognition of the severity of the problem and the AMR NAP for Pakistan contained the vision that no Pakistani citizen should suffer as a result of AMR in the coming years. The mission statement encompasses having a functional, coordinated, collaborative and sustainable AMR containment system using the ‘One Health’ approach which is aligned with the WHO GAP on AMR. The strategic priorities of the Pakistan NAP^5^ include developing and implementing a national awareness-raising and behavioural-changing strategy on AMR; establishing an integrated national AMR surveillance system; improving prevention and control of all infections; updating and enforcing regulations for human and veterinary antimicrobial use; phasing out use of antimicrobials as growth promoters; integrating AMR in all public health research agendas including vaccine research; and estimating the health and economic burden of AMR for decision making.^5^ The Pakistan chapter of the Alliance for the Prudent Use of Antibiotics (APUA, now merged with International Society of Antimicrobial Chemotherapy) states that AMR problems in Pakistan stem from lack of: access to appropriate antimicrobial therapy; regulation in human use; regulation in agricultural use, surveillance of antibiotic use and resistance; updated antibiotic use and treatment guidelines; continuing medical education on antibiotic use for prescribers; and microbiological laboratory capacity/laboratory training/diagnostic tools. They also note that AMR complicates the treatment of infectious diseases such as malaria, tuberculosis, typhoid, acute respiratory tract infections (RTIs), and diarrhoeal diseases.^6^

## Burden of disease and antibiotic use

It has been well recognized that the threat of AMR in developing countries, such as Pakistan, where a proportion of the population has limited access to education and healthcare, is significant.^7^ In common with the rest of the world, there has been a reported upsurge in infections due to resistant strains in Pakistan. For example, a 2019 WHO report ranked Pakistan among the top five countries with the highest number of neonatal deaths caused by resistant bacteria.^8^

Pakistan is the third-highest antibiotic-consuming country among low- and middle-income countries (LMICs). Between 2000 and 2015, the number of defined daily doses (DDDs) antibiotics increased by 65%, to a level of consumption of around 20 DDDs per 1000 inhabitants per day in 2015. This placed Pakistan in the mid-table position of 34th in terms of antibiotic consumption, in a study of 71 countries.^9^ When the consumption of cephalosporins and fluoroquinolones was analysed for the 5 year period 2014 to 2018, a significant increase (65%) in consumption of cephalosporins included in the WHO ‘Watch’ group of antibiotics was noted, while the population treated with ‘Reserve’ cephalosporins doubled. Amongst the fluoroquinolones, there was a major increase in consumption of ciprofloxacin.^7^

Pakistan is a developing country where the supply of antibiotics is regulated in principle by well-established legislation and antibiotics should only be dispensed with a valid prescription written by a medical practitioner.^10^ The Drug Act of 1967 in Pakistan stated that antibiotics are not over-the-counter (OTC) medications, and the Act made their availability without a prescription illegal. Despite this, throughout Pakistan it is possible to obtain antibiotics without a legitimate prescription, generally following a simple request without any clarification or explanation.^11^ It has been suggested that in some situations, such as when there is limited access to a healthcare professional or in resource-limited settings, OTC purchase of antibiotics may be needed, but self-medication risks antibiotic misuse and can result in increasing resistance.^12,13^ A 2011 global study estimated that 9% of antibiotic sales in Pakistan took place without a prescription.^13^

## Surveillance

### National surveillance studies

Many individual surveillance studies have investigated the local prevalence of AMR in Pakistan, but unfortunately, overall comprehensive studies that would provide a national picture are lacking.^14^

### Global surveillance studies

#### SOAR

The Survey of Antibiotic Resistance (SOAR), a multinational antibiotic surveillance study, has been run in a range of countries including Pakistan since 2002. The study aims to collect and make available in published, peer-reviewed papers, antibiotic susceptibility data, specifically for *Streptococcus pneumoniae* and *Haemophilus influenzae*, the most commonly isolated respiratory pathogens in the community.^15^ Key features of the SOAR study are that it focuses only on these pathogens, and that identification and susceptibility testing are performed in an independent centralized laboratory using standardized methodology (CLSI) allowing for comparisons to be made between countries or regions and for the identification of trends over time. SOAR data is analysed based on three different breakpoints: CLSI, EUCAST dose-specific and PK/PD breakpoints.

Clinical breakpoints are cut-off MIC values used to classify microorganisms into the clinical categories susceptible (S), intermediate (I) and resistant (R) in order to assist in the prediction of the clinical success or failure of a specific antibiotic.^16^ Two international organizations define breakpoint values, namely CLSI and EUCAST. Due to variation in criteria for their definition, there are some differences between CLSI and EUCAST in the clinical breakpoint values for certain bacteria for some antibiotics and this can impact susceptibility interpretation of clinical isolates.^17^ EUCAST breakpoints are dose-specific and use the EMA-approved doses that are included in the Summary of Product Characteristics of an antibiotic. This means that by application of breakpoints for higher doses, the effect of using a raised dose on the clinical efficacy of a particular antibiotic can be predicted. Currently, clinical microbiology laboratories in Pakistan use CLSI breakpoints, however the international application of the EUCAST breakpoints is expanding^18^ so it is possible that dose-specific breakpoints could also be applied in Pakistan in future. The EUCAST dose-specific breakpoints can also be used retrospectively to calculate the susceptibility of previously collected isolates to show the susceptibility levels that would have been achieved at higher doses.

Use of the EUCAST dose-specific breakpoints shows the effect of increasing the antibiotic dose on the susceptibility of a pathogen, providing additional information so the prescriber can decide if a higher dose would be of benefit. For example, *S. pneumoniae*, the most isolated respiratory pathogen^19,20^ for clinical indications such as CAP, AOM and ABRS, has over time become less susceptible to amoxicillin/clavulanic acid in some countries^21^ since the MICs of some isolates have increased. When treating infections, it is important to be able to eradicate bacterial pathogens with raised MICs to optimize clinical outcome while at the same time minimizing the risk of selecting variants with even higher MICs. This is possible because β-lactams, unlike many other antibiotics, have time-dependent killing properties. Their efficacy depends on the amount of time the drug concentration is present at the site of action. Although increasing the concentration at the infection site over a particular concentration will not have any effect on the efficacy, the use of higher doses and/or more-frequent dosing allows for successful eradication of pathogens with higher MICs because the time above the MIC is increased.^22^

**Figure 1.**
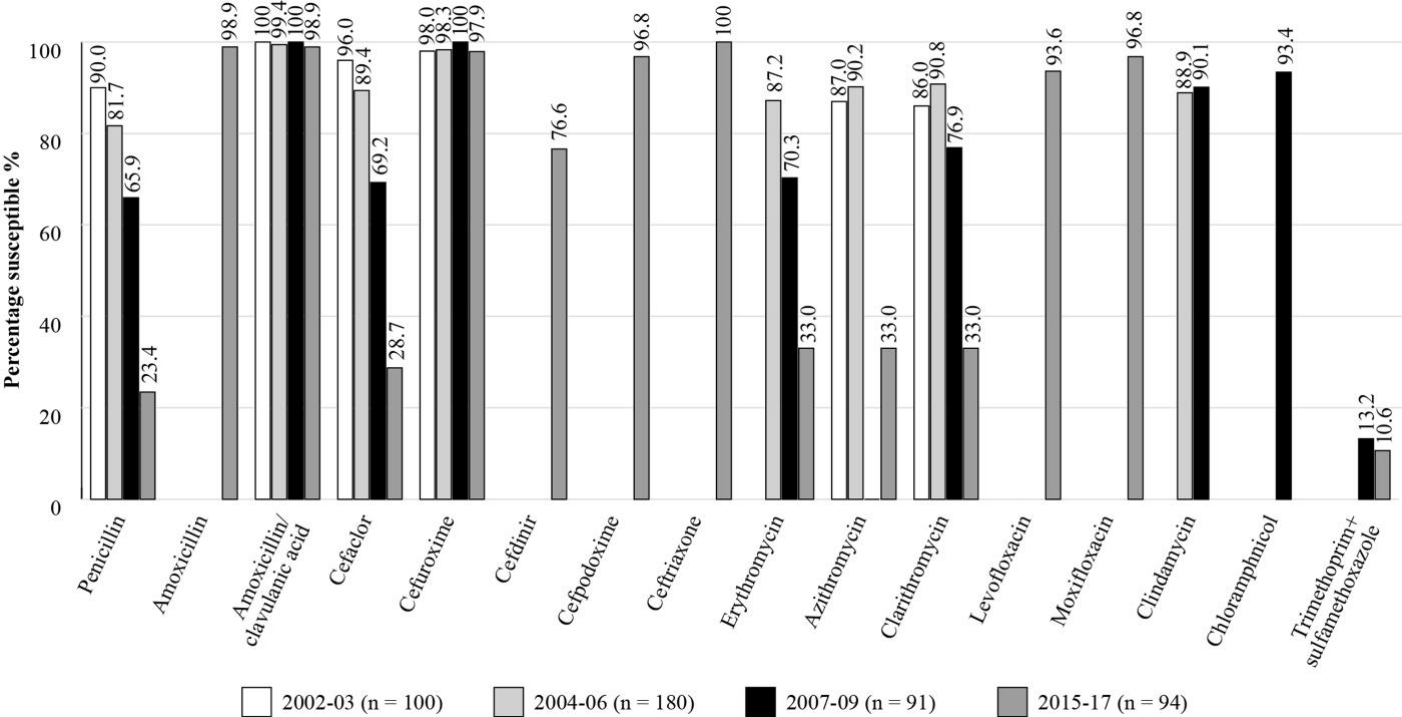
Percentage susceptibility rates based on CLSI breakpoints for antibiotics against *S. pneumoniae* isolates collected as part of the SOAR studies in Pakistan 2002–17.

Figure 1 shows susceptibility results for *S. pneumoniae* in Pakistan over four different time periods from 2002 until 2017. These data suggest that whilst the susceptibility of *S. pneumoniae* isolates remains high to amoxicillin, amoxicillin/clavulanic acid, cefuroxime, cefpodoxime, ceftriaxone and moxifloxacin, for penicillin and cefaclor, the susceptibility decreased with time so that in the 2015–17 study, the levels of susceptibility were 23.4%, 28.7%, respectively. Susceptibility for the macrolide antibiotics also decreased so that by 2015–17 the level was 33% ^15,23^

**Figure 2.**
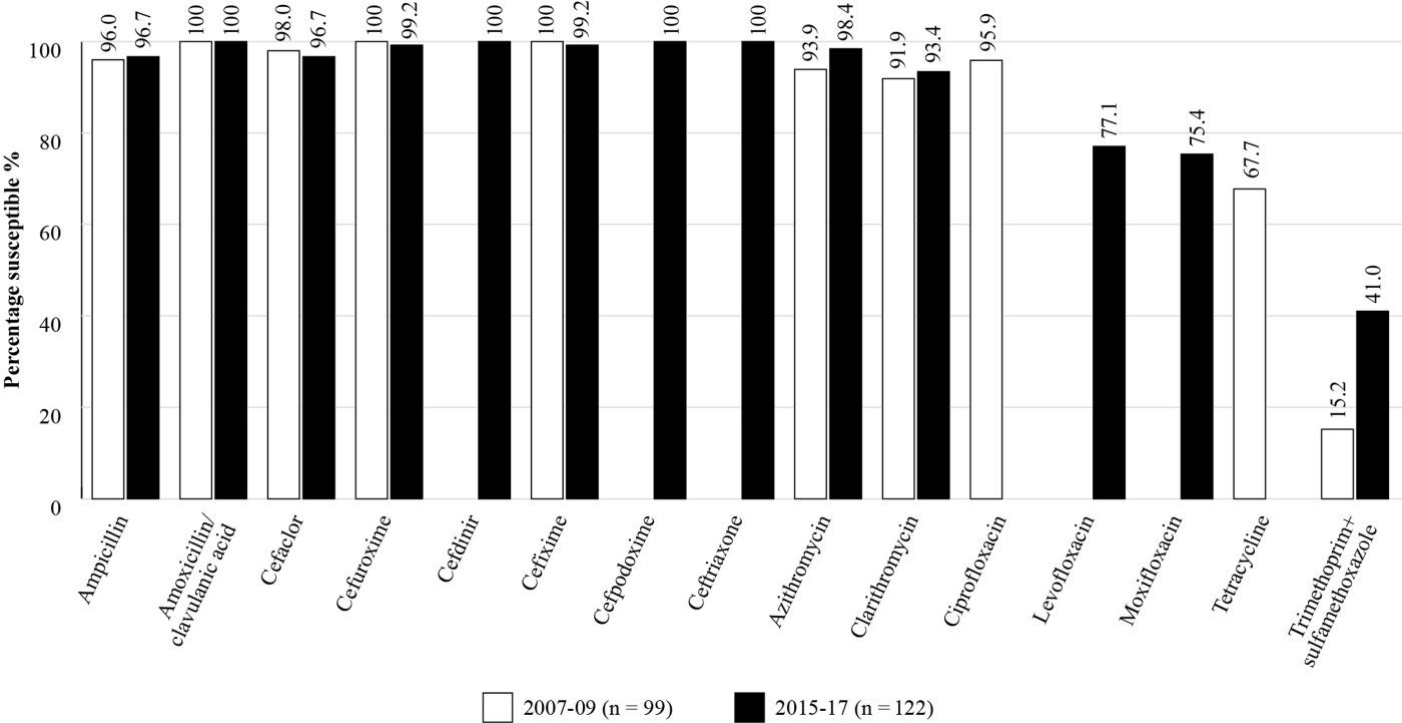
Percentage susceptibility rates based on CLSI breakpoints for antibiotics against all *H. influenzae* isolates collected as part of the SOAR study over the 10 year period in Pakistan 2007–17.

*H. influenzae* isolates collected in Pakistan in 2007–17 remained highly susceptible to most of the antibiotics tested (Figure 2). During 2015–17, applying CLSI breakpoints, susceptibility to amoxicillin/clavulanic acid was 100% but was lower for levofloxacin and moxifloxacin (77.1% and 75.4%, respectively) and for trimethoprim/sulfamethoxazole susceptibility was 41.0%.

#### ATLAS

The Antimicrobial Testing Leadership and Surveillance (ATLAS) database is a global AMR surveillance programme which is fully accessible and covers susceptibilities of a range of bacterial and fungal pathogens to a bank of antimicrobials with reference to the different breakpoints.^24^ Susceptibility data for Pakistan in CA-RTIs are available for a very small number of *S. pneumoniae* isolates and *H. influenzae* isolates from 2016 (*n *= 12) and 2017 (*n *= 8). ATLAS data is analysed based on CLSI and EUCAST breakpoints.

#### GLASS

In 2015, WHO launched the Global Antimicrobial Resistance and Use Surveillance System (GLASS). GLASS is a global system that collects national AMR data for selected bacterial pathogens that cause common infections. The aim is to monitor the prevalence of AMR among major pathogens in clinical settings^25^ to provide the supporting data required to ensure that countries can design cost-effective, evidence-based AMR response strategies. During the first four years, 91 countries/territories enrolled in GLASS and data for over two million patients from 66 countries are included.^26^ Pathogens currently included in GLASS-AMR are: *Acinetobacter* spp., *Escherichia coli, Klebsiella pneumoniae, Neisseria gonorrhoeae, Salmonella* spp., *Shigella* spp., *Staphylococcus aureus*, and *S. pneumoniae.* GLASS data is analysed based on CLSI and EUCAST breakpoints. A new and important component is the inclusion of antimicrobial consumption (AMC) surveillance at the national level.^27^

Pakistan started AMR surveillance with enrolment in GLASS in 2017. In 2019, the National Surveillance System was established, the primary goal of which is to generate evidence on the burden of AMR amongst priority pathogens. According to the May 2021 report, 40 surveillance sites are participating in the national surveillance system, these sites include 19 hospitals, one outpatient facility and 20 in/outpatient facilities.^27^

## Disease Management Guidelines

For management of the common RTIs, CAP, AOM and ABRS in Pakistan, clinicians use several country-specific local antibiotic prescribing guidelines plus a range of international guidelines as shown in Table 1. Most guidelines suggest a first-line antibiotic or antibiotics along with alternative(s) and then a second-line antibiotic or antibiotics, also with an alternative(s). The first-line antibiotic is the recommended first choice that should be prescribed by the clinician following diagnosis of the infection, supported by the criteria defined by the organization or committee; alternative(s) may be provided for use in particular circumstances. For example, if the first-line antibiotic is a β-lactam antibiotic then alternative suggestions will be for use in the case of penicillin allergy. The second-line antibiotic is for use if the first-line antibiotic does not achieve the anticipated outcome, and again alternative(s) may be included for use under specific circumstances.

**Table 1.** Examples of local and international antibiotic prescribing guidelines referred to by physicians in Pakistan for the management of community-acquired respiratory tract infections

Local antibiotic prescribing guidelines
MMIDSP 2019: The Medical Microbiology Infectious Disease Society of Pakistan’s guidelines for antimicrobial use^28^
International antibiotic prescribing guidelines
IDSA 2007: Infectious Diseases Society of America. Guidelines on the Management of Community-acquired Pneumonia in Adults^29^
BTS 2009: British Thoracic Society Guidelines for the Management of Community-acquired Pneumonia in Adults: update 2009^30^
IDSA 2011 (Endorsed by AAP): The Management of Community-acquired Pneumonia in Infants and Children Older than 3 Months of Age: Clinical Practice Guidelines by the Pediatric Infectious Diseases Society and the Infectious Diseases Society of America^31^
BTS 2011: British Thoracic Society Guidelines for the Management of Community-acquired Pneumonia in Children: update 2011^32^
IDSA 2012: IDSA Clinical Practice Guideline for Acute Bacterial Rhinosinusitis in Children and Adults^33^
AAP 2013: American Academy of Pediatrics. The Diagnosis and Management of Acute Otitis Media^34^
IDSA 2019: Diagnosis and Treatment of Adults with Community-acquired Pneumonia. An Official Clinical Practice Guideline of the American Thoracic Society and Infectious Diseases Society of America^35^
WHO 2019: World Health Organization Model List of Essential Medicines^42^

### International antibiotic prescribing guidelines

For CAP management in adults and paediatrics, the international guidelines referred to by clinicians in Pakistan include the 2009 and 2011 British Thoracic Society (BTS) guidelines^30,32^, the 2011 and 2019 IDSA guidelines^31,35^ and additionally those from the IDSA/American Thoracic Society published in 2007.^29^ For example, a first-line antibiotic treatment recommendation for CAP management from the IDSA 2019 guideline for treating adults with no comorbidities or risk factors for MRSA or *Pseudomonas aeruginosa* is amoxicillin or doxycycline or a macrolide (if the local pneumococcal resistance is <25%) but if the patient has comorbidities, the recommendation is combination therapy with amoxicillin/clavulanic acid or a cephalosporin plus a macrolide or doxycycline, or monotherapy with a respiratory fluoroquinolone. The recommended dosage for adults with comorbidities is amoxicillin/clavulanic acid, 500 mg/125 mg given three times daily, 875 mg/125 mg or 2000 mg/125 mg both given twice daily in combination with a macrolide or doxycycline.^35^ The BTS also suggests amoxicillin/clavulanic acid, cefaclor, and macrolides as an alternative to first-line amoxicillin in children with low-severity pneumonia.^32^ For AOM management, the international guidelines referred to in Pakistan include those from the American Academy of Pediatrics (AAP)^34^ and for the management of ABRS in adults and paediatrics, the international guidelines referred to in Pakistan are from the IDSA.^33^ For ABRS in children, the IDSA recommends as initial empirical treatment first-line, amoxicillin/clavulanic acid 45 mg/kg/day twice daily; and in adults first-line amoxicillin/clavulanic acid 500 mg/125 mg three times daily or 875 mg/125 mg twice daily.^33^

### National antibiotic prescribing guidelines

Considering local Pakistan guidelines for the management of CAP, AOM and ABRS, the Medical Microbiology Infectious Disease Society of Pakistan’s (MMIDSP) Guidelines for Antimicrobial Use^28^ are commonly referred to. For AOM, the MMIDSP guidelines recommend as the preferred antibiotic, in adults amoxicillin/clavulanic acid 1 g given twice daily and in children amoxicillin/clavulanic acid 20–45 mg/kg/day every 8–12 h (the dose can be up to 80–90 mg/kg). For ABRS, the preferred antibiotic treatment in adults is amoxicillin 500 mg given three times daily or amoxicillin/clavulanic acid 625 mg given three times daily.

## Antibiotic availability

Access to antibiotics may be a challenge for patients in LMICs due to cost and insufficient government expenditure or support in this area. Unreliable drug supply may also contribute to the problem. Limited access to the most-appropriate antibiotic to treat a specific infection may result in raised mortality from treatable bacterial infections and, the use of suboptimal amounts of antibiotic facilitates resistance development and allows resistant strains to persist.^36,37^

Substandard poor-quality or falsified antibiotics promote AMR^38^ and the spread of drug-resistant infections. Since poor-quality antibiotics are unlikely to contain the full dose needed to eliminate all of the infecting pathogens, use of these will encourage resistance to develop and allow resistant strains to survive and be transmitted.^39^

The quality of medicines, specifically antibiotics, is an important consideration for countries worldwide. WHO launched a Global Surveillance and Monitoring System (GSMS) for substandard and falsified products.^39^ GSMS aims to work with WHO member states to improve the quality of reporting of substandard and falsified medical products, and, importantly, to ensure the data collected are analysed and used to influence policy, procedure, and processes to protect public health, at the national, regional and the global level. Use of substandard or falsified antibiotics not only compromises clinical outcome but also risks increased AMR. The most recent GSMS summary (2013–17) reported substandard and falsified medicines in 46 member states (including Pakistan). Antibiotics represent 16.9% of all products reported, second only to malaria drugs (19.6%).

In Pakistan, several formulations of amoxicillin/clavulanic acid currently available are mentioned as first- or second-line recommendations by the RTI management guidelines. Examples include amoxicillin/clavulanic acid 500 mg/125 mg three times daily (with either a macrolide or doxycycline) which is recommended in the IDSA 2019 guidelines for the treatment of CAP in adult outpatients with comorbidities.^35^ The same recommendation is made in the IDSA 2012 guidelines as an initial empirical treatment for rhinosinusitis in adults and additionally by the MMIDSP’s Guidelines for Antimicrobial Use^28^ for rhinosinusitis in adults.

In terms of management of infection in children, amoxicillin/clavulanic acid 45 mg/kg/day twice daily is recommended by IDSA 2012 as an initial empirical therapy for rhinosinusitis.^33^

## Local insights

### Clinical microbiologist expert comment

In Pakistan, public health amenities such as clean drinking water, proper sanitation, and infection control practices are mostly lacking which not only gives rise to an increase in the incidence of communicable diseases but also increased antibiotic consumption and a resulting rise in resistance. Most classes of antibiotics are sold OTC, which often leads to misuse and overuse. Sale of counterfeit drugs and use of broad-spectrum antibiotics without the appropriate indication are also very common. In addition, Pakistan lacks good quality diagnostic facilities and credible data that would help ascertain the scale of prevailing AMR. Lack of some, or all, of the following factors leads to increasing AMR: access to appropriate antimicrobial therapy; regulation of antibiotic use in humans, agriculture and livestock; surveillance of antibiotic use and resistance; updated antibiotic use and treatment guidelines; continuing medical education on antibiotic use for prescribers; microbiological laboratory capacity and training; and diagnostic tools. Increasing AMR complicates the management of treatable infectious diseases such as malaria, tuberculosis, acute RTIs, and diarrhoeal diseases.

The prevailing conditions in Pakistan have already led to the emergence of carbapenem-resistant organisms along with the ceftriaxone-resistant *Salmonella typhi*. Emergence of microbes developing resistance against last resort antibiotics is a major health challenge. Positive gains could be made if practices were modified with the prudent use of antibiotics including using narrow-spectrum antibiotics and recommending the right antibiotic at the right dose, at the right time and for the right duration. Monitoring antibiotic misuse in livestock and agriculture is also important.

It is difficult to ascertain the burden of infectious diseases in a country as large as Pakistan, especially with a lack of local surveillance studies, however a 5 year audit (published in 2018) conducted at a tertiary care hospital in Karachi, Pakistan gives a snapshot of the commonly occurring infectious diseases^40^ highlighting the main burdens caused by malaria, tuberculosis, RTIs (upper and lower), and diarrhoeal diseases.

Surveillance of AMR of locally prevalent microorganisms is also lacking in Pakistan and would be required to guide an infection prevention and control programme, which ultimately would help in the reduction and spread of multiresistant organisms. Surveillance studies that monitor antibiotic consumption can further guide improvements in antibiotic use and ease of availability, provided they are used judiciously. In 2015, WHO launched GLASS, the first global collaborative effort to standardize AMR surveillance. However, it has been challenging for NIH Pakistan to recruit centres to participate, and more focused, localized as well as disease-specific surveillance studies are also required.

There is also a need for a unified and nationally endorsed set of treatment guidelines for communicable diseases, such as upper and lower RTIs along with gastrointestinal infections. The MMIDSP management guidelines for adults as well as children were released in 2019, but dissemination and acceptance still needs full realization. Although international treatment guidelines exist and are already in use in various centres around Pakistan, they are not tailor-made for local settings, especially with the variable prevalent resistance patterns. Moreover, many antibiotics are not available in Pakistan, which makes adherence to the international guidelines more difficult.

The main daily challenges faced within the clinical microbiology laboratory during AST reporting to a physician include explaining why results of certain antibiotics were withheld and, importantly, when the organism is pan-resistant and there are no alternative antibiotics to offer. Additional challenges include explaining why it is better to aim for a narrow- rather than a broad-spectrum antibiotic. Possible solutions to bridging communication between clinical microbiologists and physicians could be to discuss results by telephone or on the clinical ward rounds and to have orientation sessions for physicians concerning microbiology and infectious diseases. In addition, online forums could be useful for posting questions and responses.

### Clinician expert comment

Antibiotic prescribing practices are inconsistent in Pakistan. The reasons for this are multiple and include the quality of undergraduate medical education, a lack of opportunities for postgraduate training, the unregulated and fragmented healthcare system, poor diagnostics, the scarceness of quality microbiology laboratories, (hence non-availability or underutilization of culture and AST) unrestricted OTC antibiotic availability, extensive veterinary use and unregulated waste disposal into natural bodies of water. All of these factors contribute to the relentless upward trajectory of AMR and poor patient outcomes.

Antibiotic susceptibility data are available from a few, select institutions in Pakistan, on an annual or biannual basis, on a website created for this purpose (Pakistan Antimicrobial Resistance Network).^41^ The website gives extremely useful information on laboratory diagnostics and susceptibility of major bacterial pathogens in Pakistan and is a useful resource for guiding empirical and definitive therapy for various conditions.

Guidelines on the use of antibiotics released by MMIDSP are in the process of revision.^28^ The Society, through its various academic and awareness activities, has been actively involved in imparting basic knowledge about antibiotic choice and use in different clinical situations. Prominent MMIDSP members are leading infection, prevention and control (IPC) and AMR activities in Pakistan and the Society also aims to engage pharmacists to promote antibiotic stewardship activities.

Clinical guidelines are generally useful. However, in the context of Pakistan, one should be cognizant of the local susceptibility data before ‘adopting’ the guidelines in totality. The logical process would be that of ‘adaptation’ of guidelines based on local disease spectrum and susceptibility patterns.

To optimize use of antibiotics in Pakistan, with the objectives of improving patient outcomes, reducing cost and preventing rising AMR, the approach has to be multipronged. In addition to improving training of doctors (and allied healthcare professionals), microbiology laboratory systems have to be strengthened. There has to be a sustained move towards establishing aetiology early in the course of infection, rather than consistently relying on prolonged empirical use of broad-spectrum antibiotics. Susceptibility data should be made easily available to general practitioners along with instructions on how to interpret and use the data appropriately for the benefit of our patients, both in terms of cost and outcomes.

## Conclusions

In an era of rising AMR throughout the world, this paper aims to define areas where action is required to address AMR by analysing and understanding the current situation within Pakistan. Information is presented for Pakistan concerning antibiotic use and prescribing, approach to AMR, availability of local susceptibility data, use of international and/or local management guidelines, and how these link to antibiotic availability. To our knowledge this is the first time this information has been reviewed and presented in detail by country.

Antibiotic use in Pakistan is extremely high and although legislation exists prohibiting the OTC purchase of antibiotics, this continues to be a major problem. In terms of surveillance, there are a small number of studies from individual units or hospitals which have provided information on local AMR. Several global surveillance studies also include data for Pakistan (SOAR and ATLAS). The WHO GLASS study will also provide useful data in future. The surveillance data that do exist show a trend towards decreasing susceptibility amongst the common respiratory pathogens for some antibiotic classes such as the macrolides and oral penicillin, but amoxicillin/clavulanic acid and fluoroquinolone have so far retained their activity, although guidelines and regulatory bodies urge caution with the fluoroquinolones, restricting their use to limited situations due to serious safety concerns.

For management of the common RTIs in Pakistan, clinicians make use of several country-specific local antibiotic prescribing guidelines plus a wide range of international antibiotic prescribing guidelines. For the management of CA-RTIs, amoxicillin/clavulanic acid is a commonly recommended antibiotic amongst both international and local prescribing guidelines, and its choice is confirmed by currently available susceptibility data.

The clinical microbiologist and clinician view in Pakistan is in alignment in the wish for local up-to-date susceptibility data to support empirical antibiotic prescribing, since national data for a country as large as Pakistan is unlikely to be representative of the local situation. An increase in local surveillance studies would lead to the development of regularly updated management guidelines which, if applied along with updated guidelines, for example those relating to the management of COVID-19, would improve clinical outcomes and minimize further rises in AMR. A more standardized inclusive approach is needed to develop local Pakistan-specific guidelines. These guidelines would be based on up-to-date surveillance data of isolates from community-acquired infections which would make them more locally relevant for clinicians, reiterating the Consensus Principles as described in the introductory paper to this Supplement.^2^ This would pave the way for improved adherence and a higher level of appropriate antibiotic prescribing in CA-RTIs, which could, in turn, potentially limit AMR development and improve clinical outcomes for patients.
